# Argentatin Content in Guayule Leaves (*Parthenium argentatum* A. Gray)

**DOI:** 10.3390/plants12102021

**Published:** 2023-05-18

**Authors:** María Mercedes García-Martínez, Beatriz Gallego, Guayente Latorre, María Engracia Carrión, Miguel Ángel De la Cruz-Morcillo, Amaya Zalacain, Manuel Carmona

**Affiliations:** 1 Instituto Técnico Agronómico Provincial de Albacete (ITAP), Parque empresarial Campollano, 2ª Avenida, 61, 02007 Albacete, Spain; mariamercedes.garcia@uclm.es; 2 E.T.S.I. Agronómica, de Montes y Biotecnología (ETSIAMB), Cátedra de Química Agrícola, Universidad de Castilla-La Mancha, Avda. de España s/n, 02071 Albacete, Spain; guayente.latorre@uclm.es (G.L.); amaya.zalacain@uclm.es (A.Z.); 3 Instituto de Toxicología de la Defensa, Hospital Central de la Defensa Gómez Ulla, Gta Ejército 1, 28047 Madrid, Spain; bgaligl@mde.es; 4 Institute for Regional Development (IDR), Food Quality Research Group, Universidad de Castilla-La Mancha Campus Universitario s/n, 02071 Albacete, Spain; mengracia.carrion@uclm.es (M.E.C.); miguelangel.cruz@uclm.es (M.Á.D.l.C.-M.)

**Keywords:** guayule leaves, argentatin A, isoargentatin A, argentatin B, isoargentatin B

## Abstract

Approximately one-third of the waste biomass from the cultivation of guayule (*Parthenium argentatum* A. Gray) for natural rubber production is leaf tissue; however, whether it can be valorized is not known. Guayulins and argentatins are potential high-value products that can be recovered from guayule resin during rubber/latex processing. Argentatins are highly abundant in guayule stem resin; however, unlike the guayulins, their occurrence in leaves has not been investigated. The present study determined the content of argentatins and isoargentatins A and B in the leaves of a pure guayule accession (R1040) and two hybrids (CAL-1 and AZ-2) under conditions of irrigation and non-irrigation. The resin content in leaves was ~10%, which provides a suitable starting point for economic exploitation. The highest production of argentatins occurred in plants under irrigation, with yields of 4.2 and 3.6 kg ha^−1^ for R1040 and AZ-2, respectively. The R1040 accession had the highest percentage of resin and the greatest total argentatin content (24.5 g kg^−1^ dried leaf), principally due to the abundance of argentatin A. Contrastingly, CAL-1 consistently showed the lowest argentatin content based on dried leaf weight and production (0.6 kg ha^−1^). The substantial abundance of argentatins in guayule leaves suggests the potential for future exploitation.

## 1. Introduction

Guayule (*Parthenium argentatum* A. Gray) is the best candidate for countries consuming large quantities of rubber with arid and semi-arid agricultural areas where it is not possible to cultivate *Hevea brasiliensis* [1]. It was used as a source of rubber in the past, especially for the manufacturing of tyres; however, it is now estimated that it could only become an economically competitive crop again if all its co-products were exploited, rather than just rubber [2,3,4].

Approximately 30% of the waste biomass from the cultivation of guayule is leaf tissue [5], which is typically discarded prior to bulk rubber extraction [2]. This process occurs because leaf materials can reduce rubber recovery due to the formation of very fine particles that clog the filtration systems and increase the ash content associated with rubber degradation and quality reduction [2,6]. The complete morphological characterization of guayule leaves was performed within 27 guayule accessions in Spain, grouping them into three main groups depending on their morphological parameters, which, in general, were apiculate leaves with the petiole delimited based on leaf margin, serrate or lanceolate types [7]. Differences identified within the recent USDA germplasm collection confirm that growth environment has a major influence on leaf morphological characterization [8].

Other studies proposed the valorization of different plants from their leaves [9,10,11,12]. Early studies on the valorization of guayule by-products focused on the extraction of essential and volatile oils from stem resin [13,14], while more recent attention was paid to phenolic compounds [7,15,16], particularly hydroxycinnamic and hydroxybenzoic acids, flavones, flavanols and anthraquinones [7,15]. In addition to the stem resin, a significant proportion of total guayulin content is found in leaf tissue, where the four major guayulins (A–D) are observed in a more consistent relationship than in the stem [17], whereas rubber content in the leaves is discarded [18].

**Figure 1:**
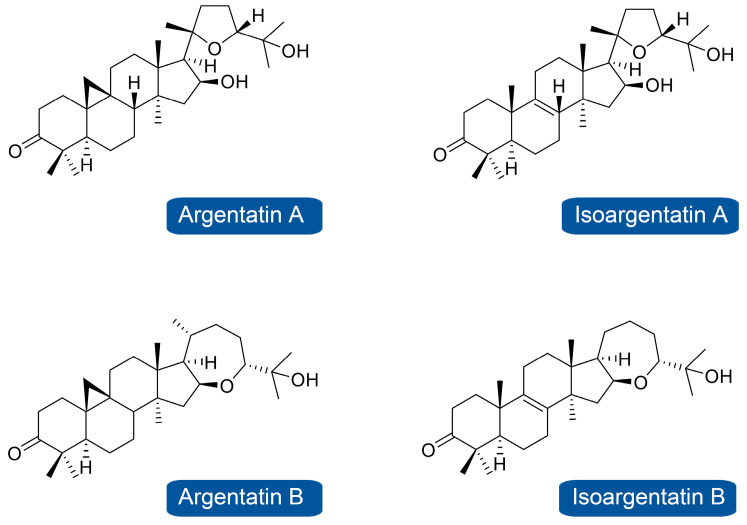
Structure of most abundant argentatins and isoargentatins in guayule accessions [19].

Beyond the commercialization of the guayulins, a large number of other extractable secondary metabolites that could be economically exploited are present in relevant amounts in guayule stem resin, including triterpenes and their derivatives, known as the argentatins. Recently, Gallego et al. [19] identified, for first time, the argentatins and derivates profile in 27 different guayule accessions with LC-CR-MS technology (Orbitrap^®^). There is limited information on argentatin accumulation as their routine quantification is challenging because the structure lacks double bonds and shows a weak chromophoric functionality; however, Gallego et al. [20] recently proposed their quantification using a refractive index detector after their chromatographic separation. The most abundant argentatins in guayule resin correspond to argentatin A (C_30_H_48_O_4_) and B (C_30_H_48_O_3_), as representative of the cycloartenol-type triterpenes, and isoargentatin A (C_30_H_48_O_4_) and isoargentatin B (C_30_H_48_O_3_), with lanostane-type triterpenes (Figure 1).

Argentatins attracted considerable attention, in particular argentatins A and B, because they have several biological activities that make them potentially useful as pharmacophores. Argentatin A has antimicrobial activity against pathogenic bacteria [21], and argentatins A, B and D have anti-inflammatory activity [22]. In vitro cytotoxic activity against a broad panel of human cancer cell lines was previously attributed to argentatin A and B [22,23,24,25], and in vivo in a mouse xenograft model of human colon cancer was previously associated with argentatin A [25]. More recent studies explored the use of argentatins as precursors for the synthesis of compounds with enhanced activity and lower toxicity [26,27,28].

The production of argentatins in guayule stems depends on the accession used and the time of harvest, with the spring and summer periods showing maximum accumulation [20]. To the best of our knowledge, no previous study examined whether argentatins are produced in guayule leaves. Therefore, the aim of the present study was to explore the content of the four major argentatins (argentatins and isoargentatins A and B) in the leaves of three guayule and hybrid accessions under different agronomic conditions (irrigation and non-irrigation). The content of argentatins in leaves was then compared with the content in stems in the same accessions.

## 2. Results

### 2.1. Leaf Resin Extraction and Quantitation of Argentatins

The leaf resin content from the three guayule accessions ranged from 9.5% to 11.5% (Table 1), and the standard deviation range for all accessions was very small, with the exception of AZ-2 under non-irrigation conditions. No significant differences in leaf resin content were found between the three accessions under irrigation conditions (Table 1); however, the resin content of R1040 was significantly greater under non-irrigation than irrigation conditions and was also significantly greater than that of the other two accessions.

In relation to total argentatin content (aT), the R1040 accession showed the highest total content relative to AZ-2 and CAL-1 (R1040 > AZ-2 = CAL-1) under both agronomic conditions, due to its significantly greater content of aA. Under non-irrigated conditions, aT content remained highest in R1040, and was even greater than under irrigated conditions. Contrastingly, the aB content was greater in AZ-2 and CAL-1 hybrids than in R1040 under irrigation conditions, whereas no significant differences were observed under non-irrigation conditions (Table 1).

### 2.2. Argentatin Production per Hectare

Overall, the leaf yield (per hectare) was higher with irrigation than without, with a mean yield of 215.6 kg ha^−1^ for irrigated plots of the three accessions and 74.9 kg ha^−1^ for non-irrigated plots (Table 2). When irrigated, R1040 produced a greater leaf yield per hectare (258.4 kg), but both AZ-2 and R1040 produced the highest total argentatin yield (kg ha^−1^) when considering the content of argentatins (g kg^−1^ dried leaf). Likewise, AZ-2 and R1040 produced the highest content of aA and isoaA. No significant differences were observed in the production of aB between the three accessions.

## 3. Discussion

The leaf resin content from the three guayule accessions studied is similar to the values reported for stem resin content by Gallego et al. [20], and is a suitable starting point for its industrial exploitation. Moreover, the resin percentage was consistent with values reported by Dehghanizadeh et al. [2] and Rozalén et al. [17], who obtained a yield of 9–10% in plants of the same age, and was much greater than the 4% reported in older plants (15–18 years old) by Spano et al. [29]. No significant differences in leaf resin content were found between the three accessions under irrigation conditions; however, R1040 resin content was clearly highest under non-irrigation conditions.

In relation to total argentatin content (aT), R1040 and AZ-2 accessions produced the highest content under both agronomic conditions. Analysis of all argentatins and isoargentatins revealed two distinct profiles: the content of aB was greater than aA in CAL-1, whereas the opposite was seen for AZ-2 and R1040. In contrast, the content of isoaA and isoaB was maintained for all three accessions (isoaA > isoaB) under both agronomic conditions.

In terms of leaf production per hectare, irrigation yields a higher content than non-irrigation. Through comparing the results of argentatins and isoargentatins obtained in leaves with their content in guayule stems [19], it is evident that the values obtained for the same accessions by Gallego and colleagues are much higher. Nonetheless, if the same accessions are compared with the stems of plants of the same age and under the same conditions [20], the contents of aA and aB are very similar.

The argentatin content in guayule leaves ranged between 1.2 and 2.7% dry weight, which is a credible concentration for a bioactive compound in a vegetable by-product with proven antitumor activity [25]. The semi-synthesis of argentatins A–C analogs was also successfully performed in an effort to increase their cytotoxicity [26]. Notably, the production of new drugs is largely (64%) derived from natural products, generally via semi-synthesis [30,31]. For example, podophyllotoxin is the starting material for the important antineoplastic drugs etoposide and teniposide. Podophyllotoxin is found in a concentration of ~1.3% in the rhizome of Podophyllum hexandrum [31,32]. Another example is paclitaxel, traded under the brand name Taxol, which is the most successful anticancer phyto-drug developed in the last 50 years. The content of the paclitaxel precursor in Taxus baccatta (European Yew) is even lower than that of podophyllotoxin, being around 0.01–0.03% [33]. It should be noted that the content of argentatins in leaves is much higher than other compounds that have been considered to valorize guayule leaves, including phenolic compounds (0.8%, as reported by Jara et al. [7] or their essential oil content, which were reported to be 1.04% [13].

## 4. Conclusions

A resin content of almost 10% in leaves is a relevant amount for its commercial exploitation, either directly for raw resin applications or for the isolation of compounds of interest, such as the argentatins. In the present study, the highest total argentatin production was achieved with R1040 and AZ-2 accessions under irrigation, together with the highest production of aA and isoaA. The production of aB and isoaB can be achieved indistinctly under irrigation conditions with the three tested accessions.

## 5. Materials and Methods

### 5.1. Guayule Samples

The pure R1040 guayule accession and the CAL-1 and AZ-2 hydrids were selected for study under irrigated and non-irrigated conditions. The experimental plot (0.3 ha) was planted in May 2019 in Santa Cruz de la Zarza, Toledo, Spain. A randomized complete block design with four replicates per accession was utilized for analysis. A sample of three adjacent plants was taken for each of the four replicates, giving a total of twelve plants from each accession. Plants (24 months from sowing) were manually harvested and packaged into kraft bags. Whole plants were dried for 48 h at 60 °C to achieve a moisture content of ~12%. Leaves and stems were then manually separated, and the dry biomass weight was calculated from each fraction. Leaves and stems were grounded using the following two-step procedure: in the first step, the material (30 g) was ground into 2-mm-sized particles using a hammer grinder (Mader 57075, Mealhada, Portugal) for 10–15 s; and in the second step, the material was ground into particles of 0.5 mm using a centrifuge grinder (Retsch ZM 1000, Haan, Germany) for 2 min. Dried ground samples were stored in screw cap-closed glass vessels at room temperature. The moisture content of the samples was measured using a halogen lamp moisture balance, model XM-120T (Cobos, Barcelona, Spain) at 105 °C. When moisture loss was less than 0.1% in 180 s, the material was considered to have reached a constant mass.

### 5.2. Resin Determination

Extraction of the resin fraction was performed in an ASE E-914 Speed Extractor (BUCHI, Postfach, Switzerland), as described by Rozalén et al. [34]. Resin extracts were collected in 240-mL flasks and transferred to pre-weighed flasks equilibrated in a desiccator for 30 min. Solvent evaporation was performed in a Multivapor BUCHI P-6 (Postfach, Switzerland) parallel system at 150 mbar and 50 °C. After evaporation, the pre-weighed flasks were stored for 60 min in a desiccator before final weighing. The resin percentage was determined gravimetrically through considering the dry weight. Each sample was twice extracted.

### 5.3. Argentatins Determination via HPLC-RID

Twenty microliters of resin dissolved in ethanol at 10 mg mL^−1^ and filtered (0.22 µm) was injected into an Agilent 1200 high-performance liquid chromatography (HPLC) system (Agilent Technologies, Palo Alto, CA, USA) equipped with a refractive index detector (RID) (Agilent, G1362A). Separation was performed on a reverse-phase ACE Excel 3 C18-PentaFluorPhenyl column (150 × 4.6 mm, 3-μm particle size) protected with an ACE Excel HPLC Pre-column Filter (0.5-μm particle size) (both from Advanced Chromatography Technologies Ltd., Reading, Berkshire, UK). An isocratic program was used with acetonitrile (60%) and Milli Q-grade water (40%) as solvents. The analysis time was 60 min. Agilent ChemStation software (version B.03.01) was used for quantification of argentatins and isoargentatins A and B (aA, isoaA, aB, isoaB), using the aA (95.9% purity) and aB (92.4% purity) standards kindly provided by Dr. Mariano Martínez-Vázquez (Universidad Nacional Autónoma de México). Quantification of argentatins using the RID was carried out from the corresponding six-level calibration curve: aA (10–1000 mg L^−1^; r^2^ = 0.9976) and aB (50–1000 mg L^−1^; r^2^ = 0.9980).

## Figures and Tables

**Table 1:** Descriptive statistics for argentatins and isoargentatins A and B content in leaves under different conditions of irrigation.

Crop System	Accession	Statistical Parameters	Resin Yield (%)	Samples Analyzed via RID (g kg^−1^ Dried Leaf)				
				aA	isoaA	aB	isoaB	aT
Irrigated	AZ-2	Min.	9.14	6.86	2.52	6.33	0.67	
		Max.	9.96	6.98	2.57	6.55	0.69	
		Mean	9.55 A, a	6.92 A, a, δ	2.54 A, b, β	6.44 A, b, γ	0.68 B, b, α	16.58 A, a
		SD	0.58	0.08	0.04	0.16	0.01	
	CAL-1	Min.	9.18	6.02	1.25	6.78	0.54	
		Max.	9.85	6.47	1.35	7.33	0.57	
		Mean	9.52 A, a	6.25 B, a, γ	1.30 A, a, β	7.05 A, b, δ	0.56 A, a, α	15.16 B, a
		SD	0.48	0.32	0.07	0.38	0.02	
	R1040	Min.	9.44	9.50	2.63	5.44	0.64	
		Max.	9.97	9.91	2.82	5.64	0.67	
		Mean	9.71 A, a	9.70 A, b, δ	2.73 A, b, β	5.54 A, a, γ	0.65 A, b, α	18.62 A, b
		SD	0.38	0.29	0.13	0.14	0.02	
Non- irrigated	AZ-2	Min.	8.28	7.50	1.76	5.37	0.38	
		Max.	10.79	9.74	2.25	7.04	0.47	
		Mean	9.53 A, a	8.62 A, b, δ	2.00 A, a, β	6.21 A, a, γ	0.43 A, a, α	17.26 A, b
		SD	1.77	1.59	0.35	1.18	0.07	
	CAL-1	Min.	9.80	3.79	1.37	5.99	0.50	
		Max.	9.98	3.83	1.40	6.39	0.52	
		Mean	9.89 A, a	3.81 A, a, γ	1.38 A, a, β	6.19 A, a, δ	0.51 A, a, α	11.89 A, a
		SD	0.13	0.03	0.02	0.28	0.01	
	R1040	Min.	11.15	13.03	3.19	6.58	0.85	
		Max.	11.92	13.78	3.53	7.10	0.92	
		Mean	11.53 B, b	13.41 B, c, δ	3.36 B, b, β	6.84 A, a, γ	0.88 B, b, α	24.50 B, c
		SD	0.54	0.53	0.24	0.37	0.05	

Note: aA, argentatin A; isoaA, isoargentatin A; aB, argentatin B; isoaB, isoargentatin B; aT, total argentatins and isoargentatins A and B; RID, refractive index detection; SD, standard deviation. Different letters indicate significant differences between different crop system and accessions at 95% level of confidence. Uppercase letters refer to an ANOVA comparing differences between crop systems for same accessions; lowercase letters refer to an ANOVA comparing differences between accessions within same crop system; greek letters refer to an ANOVA comparing differences between argentatins within same accession.

**Table 2:** Leaf yield per accession and argentatin production per hectare.

Crop System	Accession	Leaf Yield per ha (kg)	Production per ha (kg)				Total Production per ha (kg)
			aA	isoaA	aB	isoaB	
Irrigated	AZ-2	207.19 B, a	1.50 B, ab	0.55 B, ab	1.39 B, a	0.15 B, a	3.59 B, ab
	CAL-1	181.20 B, a	1.07 B, a	0.22 B, a	1.21 B, a	0.10 B, a	2.60 B, a
	R1040	258.44 B, b	2.19 B, b	0.62 B, b	1.25 B, a	0.15 A, a	4.21 B, b
Non-irrigated	AZ-2	81.20 A, a	0.73 A, ab	0.17 A, ab	0.53 A, a	0.04 A, a	1.47 A, ab
	CAL-1	50.74 A, a	0.19 A, a	0.07 A, a	0.31 A, a	0.03 A, a	0.59 A, a
	R1040	92.72 A, a	1.35 A, b	0.34 A, b	0.69 A, a	0.09 A, b	2.47 A, b

Note: aA, argentatin A; isoaA, isoargentatin A; aB, argentatin B; isoaB, isoargentatin B; aT, total argentatins and isoargentatins A and B. Different letters between rows indicate significant differences between different crop system and accessions at 95% level of confidence. Uppercase letters refer to an ANOVA that compares differences between crop system for same accessions; lowercase letters refer to an ANOVA that compares differences between accessions within same crop system.

## Data Availability

The data presented in this study are available upon request from the authors.
